# Diagnostics of Metabolic Bone Disease in Extremely Preterm Infants—Clinical Applicability of Bone Turnover Biochemical Markers and Quantitative Ultrasound

**DOI:** 10.3390/children11070784

**Published:** 2024-06-27

**Authors:** Sandra Cerar, Lara Vurzer, Aneta Soltirovska Šalamon, Lilijana Kornhauser Cerar, Matevž Trdan, Domen Robek, Tina Perme, Ajda Biček, Adrijana Oblak, Janja Marc, Darko Černe, Vanja Erčulj, Štefan Grosek

**Affiliations:** 1 Department of Neonatology, Division of Paediatrics, University Medical Center Ljubljana, 1000 Ljubljana, Slovenia; sandra.cerar@kclj.si (S.C.); aneta.soltirovska@kclj.si (A.S.Š.); 2 Department of Paediatrics, Community Health Centre Ljubljana, 1000 Ljubljana, Slovenia; larapetric@gmail.com; 3 Faculty of Medicine, University of Ljubljana, 1000 Ljubljana, Slovenia; 4 Neonatology Section, Department of Perinatology, Division of Gynaecology and Obstetrics, University Medical Centre Ljubljana, 1000 Ljubljana, Slovenia; lilijana.kornhauser-cerar@guest.arnes.si (L.K.C.); matevz.trdan@kclj.si (M.T.); domen.robek@gmail.com (D.R.); tina.perme@kclj.si (T.P.); 5 Department of Nuclear Medicine, University Medical Centre Ljubljana, 1000 Ljubljana, Sloveniaadrijana.oblak@kclj.si (A.O.); 6 Faculty of Pharmacy, Department of Clinical Biochemistry, University of Ljubljana, 1000 Ljubljana, Slovenia; janja.marc@kclj.si (J.M.); darko.cerne@kclj.si (D.Č.); 7 Clinical Institute for Clinical Chemistry and Biochemistry, University Medical Centre Ljubljana, 1000 Ljubljana, Slovenia; 8 Faculty of Criminal Justice and Security, University of Maribor, 1000 Ljubljana, Slovenia; vanja.erculj@rosigma.si; 9 Department of Paediatric Intensive Care, Division of Paediatrics, University Medical Centre Ljubljana, 1000 Ljubljana, Slovenia

**Keywords:** metabolic bone disease, bone mineral density, premature, calcium, alkaline phosphatase

## Abstract

Background: Significant improvement in neonatal care has enabled increasing survival of preterm infants. Metabolic bone disease of prematurity is often overlooked due to other comorbidities of preterm birth. The best approach is screening and prevention of the disease in high-risk infants such as preterm infants. Aim: We followed up the clinical, radiological, and serum biochemical markers of metabolic bone disease in extremely preterm infants (<28 weeks of gestation). The clinical applicability and validation of C-terminal telopeptide of type I collagen (CTX-I) as a novel bone turnover marker were assessed. Standard and novel biochemical bone turnover markers and quantitative ultrasound were compared. Method: Patients’ data were collected from medical records. Assessments of calcium, phosphate, alkaline phosphatase, bone-alkaline phosphatase, CTX-I, and quantitative ultrasound were prospectively performed twice in 42 extremely preterm infants at postmenstrual ages of 30–32 weeks and 36–40 weeks. Bone mineral density was measured by quantitative ultrasound. Conclusion: Phosphate, alkaline phosphatase, bone alkaline phosphatase, calcium, or ionized calcium are not related to gestational age, but bone mineral density, measured by quantitative ultrasound, is related. There is no correlation between standard and novel biochemical markers and quantitative ultrasound for the identification of metabolic bone disease.

## 1. Introduction 

Metabolic bone disease (MBD) of prematurity is defined by under-mineralization of the preterm infant skeleton. It is a multifactorial disease in which, in addition to immature bone formation due to prematurity, environmental, mechanical, and genetic factors are involved [1,2]. Due to preterm birth, there is a diminution of the active transplacental transport of minerals, calcium and phosphate, which may result in MBD. Reduced mineralization is represented by hypophosphatemia, hyperphosphatasemia, and late onset of a radiological finding of bone demineralization [3]. The incidence of MBD is highest in newborns born before 28 weeks of gestation with extremely low birth weight (<1000 g), occurring in 55%, and in very low birth weight (<1500 g), occurring in 23% [4,5]. Several antenatal risk factors for MBD exist, including maternal hypovitaminosis D, chronic damage to the placenta (preeclampsia, chorioamnionitis, placental infections), and a lack of mechanical stimulation owing to interruption of fetal movements against the uterus wall due to premature birth. Extremely low birth weight infants receiving prolonged (>4 weeks) total parenteral nutrition, diuretic treatment, corticosteroids, and/or methylxanthines are at increased risk of developing MBD postnatally. Common neonatal conditions such as sepsis, necrotizing enterocolitis, prolonged immobilization, bronchopulmonary dysplasia, neuromuscular conditions, and cerebral pathology also increase the risk of inadequate bone mineralization [1,3,6,7]. Preventing MBD in premature newborns involves nutritional support with adequate intake of calcium and phosphate, vitamin D supplementation, breast milk fortification, early enteral nutrition, screening and monitoring of high-risk patients, and minimizing risk factors (i.e., judicious use of diuretics and corticosteroids), and avoiding prolonged immobility. 

MBD significantly impacts the quality of life of premature newborns. The condition leads to increased fracture risk, which can cause pain, limit movement, and require medical interventions. Poor bone mineralization can lead to stunted growth and delays in motor development. Bone fragility can result in chronic pain, which affects the infant’s comfort, sleep, and overall well-being. MBD often necessitates extended hospital stays and frequent medical interventions, including diagnostic tests, treatment for fractures, and nutritional support. This can disrupt bonding and caregiving routines and increase stress for both the infant and the family. MBD in infancy can predispose individuals to long-term skeletal issues, such as lower bone mass and higher fracture risk later in life. These long-term effects can have a lasting impact on quality of life [1,3,6,7]. 

Clinical signs often remain silent until the 5th to 11th week of life, when respiratory effort is increased due to chest wall instability caused by softening or fractures of the ribs, enlargement of the cranial sutures, long bone fractures, and postnatal growth failure [3]. 

It is necessary to screen infants who are at risk of developing MBD because clinical findings appear late and diagnosis may sometimes be overlooked. In clinical practice, diagnosis depends on recognizing risk factors, biochemical markers, and quantitative bone mass measurements [3]. 

Assessment of biochemical indices of bone formation and resorption is an important methodological advance. Serum alkaline phosphatase (ALP) is an enzyme with at least four isoenzymes present in the liver, bones, kidneys, and intestine. Bone-specific alkaline phosphatase is a bone turnover marker and is located on osteoblast surfaces [3]. Products of collagen type I synthesis, including procollagen type I N-terminal propeptide and C—terminal propeptide (PINP [8] and PICP), bone alkaline phosphatase, and non-collagenous protein osteocalcin are used as bone formation markers, whereas crosslinked C-terminal telopeptide type I collagen (CTX-I) is used as a bone resorption marker [7,9]. In addition to biochemical assessment, radiological assessment of bone mineral density (BMD) is important. Although dual-energy X-ray absorptiometry (DXA) is regarded as a reference standard for assessing BMD, it has several limitations. Another possible modality for BMD assessment is quantitative ultrasound (QUS). It is easy to use, radiation-free, and has a short examination time [10]. The QUS parameter “speed-of-sound value” (SOS-value) is an indicator of BMD and correlates with gestational age [11]. The QUS modality has potential as an initial screening tool for the identification of newborns with MBD, as well as for assessing the subsequent development of bone health [12]. 

In this study, well-known postnatal risk factors and their contribution to MBD in our newborns were studied. We aimed to:

(a) Follow up prospectively the clinical, radiological, and serum biochemical markers of MBD in extremely preterm infants;
(b) Assess the clinical applicability and validation of CTX-I as a novel bone turnover marker for diagnostics of MBD;
(c) Assess the correlation between standard and novel biochemical bone turnover markers for diagnostics of MBD in comparison with quantitative ultrasound measurement data.

We hypothesized that there is a correlation between standard and novel serum biochemical bone turnover markers and quantitative ultrasound for the identification of MBD in extremely premature newborns. 

## 2. Materials and Methods

### 2.1. Study Participants 

The prospective clinical observation study included a sample of extremely premature infants, born between 23 0/7 and 27 6/7 weeks of gestation, from May 2022 to September 2023, who were admitted to NICU, University Medical Centre Ljubljana, Division of Gynaecology and Obstetrics, Ljubljana, Slovenia. The inclusion criteria were extremely low gestational age newborns, with expected comorbidities due to prematurity, such as MBD, intraventricular hemorrhage, respiratory distress, necrotizing enterocolitis, bronchopulmonary dysplasia, and persistent ductus arteriosus. Patients’ data were obtained prospectively and from medical records.

No child met the exclusion criteria: (suspected) genetic or metabolic congenital disorders and death before discharge. The study protocol was approved by the Medical Ethics Committee of the Republic of Slovenia, with protocol number 0120-503/2021/5. Written informed consent was obtained from the parents. 

### 2.2. Study Measurements

All of the infants from our observational cohort received appropriate treatment for extremely low gestational age newborns. In accordance with our nutritional strategy, subjects were introduced to early minimal enteral nutrition (in first 24–48 h if clinically stable, with milk volumes of 12–24 mL/kg/d). When enteral feeding was established, our subjects were fed exclusively with human milk, and most prematures were also fed with a human milk fortifier formula for additional protein and mineral supply required by prematures. Since the 7th day of life, after enteral feeding tolerance, prematures were supplemented with vitamin D (cholecalciferol) at 800–1000 IU per day. The daily **c**alcium intake was 150–220 mg/kg/day. We consistently followed the recommendations of ESPGAN [13] for the nutrition of critically ill children in the preparation of enteral and parenteral nutrition. The parenteral nutrition composition prescription was also guided by a computer tool. The study participants followed their growth curves for their gender, age, and gestation.

EOS refers to a bloodstream infection occurring within the first 72 h of life (up to 7 days of life). It is characterized by a systemic inflammatory response to an infection of bacterial origin. The definition of late onset sepsis refers to a bloodstream infection after the first 72 h (after 7 days) of life, often associated with hospital-acquired infections and prolonged medical interventions. SIRS is a clinical syndrome characterized by a systemic inflammatory response to a variety of severe clinical insults, which can be infectious or non-infectious (perinatal asphyxia, major surgery).

At 30–32 weeks and at term age, serum alkaline phosphatase activity (ALP), ionized calcium, phosphate, bone-specific alkaline phosphatase, and crosslinked C-terminal telopeptide type I collagen (CTX-I) levels were measured in blood. We performed quantitative ultrasound (QUS) to evaluate BMD at the same time. 

### 2.3. Serum Concentrations of Bone Turnover Markers 

C-terminal telopeptides of type I collagen degradation products (CTX-I) and bone-specific alkaline phosphatase were measured by IVD-approved immunoassay methods on an automated analyzer iSYS (Immunodiagnostic Systems GmbH, IDS, Frankfurt am Main, Germany). Blood samples without coagulant were centrifuged for 10 min at 1800× *g* and serum was used for CTX-I and bone-specific alkaline phosphatase determination. Both of the methods were routinely used in clinical practice and applied in accordance with directive 98/79/EC.

#### 2.3.1. Crosslinked C-Terminal Telopeptide Type I Collagen (CTX-I)

The method for CTX-I (CrossLaps^®^) determination was based on chemiluminescence technology and used two highly specific monoclonal antibodies against the specific amino acid sequence EKAHD-β-GGR, one of which is conjugated to biotin, the other to acridinium. The signal was directly proportional to the concentration of analyte in the original sample. The reportable range of the assay was 0.033–6.000 µg/L, with within-run and total imprecisions from 2.1 to 4.9% and from 4.7 to 8.8%, respectively. 

#### 2.3.2. Bone-Specific Alkaline Phosphatase 

The bone-specific alkaline phosphatase method (Ostease^®^ BAP) was based on spectrophotometric technology with the use of a biotin-labeled BAP-specific monoclonal antibody. The signal was directly proportional to the amount of bone-specific alkaline phosphatase present in the original sample. The reportable range of the assay was 1–75 μg/L, with within-run and total imprecisions from 1.3 to 2.0% and from 6.5 to 9.0%, respectively. Samples with values above 75 μg/L were diluted with IDS-iSYS Diluent B to reach the final concentration.

### 2.4. Laboratory Measurements 

Ionized calcium was measured in venous blood collected with lithium heparin using a Rapidlab 1265 analyzer (Siemens Healthcare Diagnostics, New York, NY, USA). After the measurement, the blood was centrifuged for 10 min at 2000× *g* and plasma concentrations of total calcium, inorganic phosphate, and alkaline phosphatase were measured using an Alinity c analyzer (Abbott, Wiesbaden, Germany). An abnormal (low) level of total calcium was set at <2.04 mmol/L and ionized calcium at <1.2 mmol/L. An abnormal (low) level of phosphate was set at <1.8 mmol/L. An abnormal (increased) level of alkaline phosphatase was set at >10 µkat/L (>600 IU/L). 

The QUS measurements was performed in all 42 patients included, while simultaneous laboratory assessment due to a sample error was made in only 39 and 41 patients, respectively. 

### 2.5. Quantitative Ultrasound Measurement of the Tibial Bone Speed-of-Sound 

Quantitative ultrasound (QUS) measures the time taken by the ultrasound signal to travel through the bone tissue between two transmitters and two receivers contained within the probe. These propagation times are used by a proprietary algorithm to determine the bone speed-of-sound (SOS), which is expressed as meters per second (m/s). SOS-value, assessed by QUS, was used as an indicator of BMD in our study. All the ultrasound measurements were performed only by one physician.

BMD was measured by a quantitative ultrasound machine, Sunlight Omnisense 7000P (Sunlight Medical Ltd., Tel Aviv, Israel), with an appropriately sized probe and software designed for pediatric populations (BonAgePediatric). After entering the child’s data, including first and last name, date of birth, gestational age, gender, birth weight, and currently measured body weight, into the software system, measurements were taken in the middle of the right tibia. A single measurement took 5–10 s. The software used the three most consistent measurements to compute the result. Results were noted as SOS and Z-score (units of standard deviations relative to the mean for age- and sex-matched population reference values).

For adults, the World Health Organization defines osteopenia as a decrease in BMD by −1 to −2.5 Z score and osteoporosis as a decrease in BMD by more than 2.5 standard deviations below the average value of the same race, age, and sex [14]. In children, the low BMD threshold is set at Z-score ≤ −2.0, with consideration for long bone fracture characteristics [15,16]. According to manufacturer’s SOS-value references and in terms of severity, BMD was categorized into two categories: 1st with Z-score (−1) to (−2.5) and 2nd with Z-score < (−2.5).

First measurements of BMD in the premature newborns were obtained in the period of 30–32 weeks of postmenstrual age and repeated at 36–40 weeks of postmenstrual age.

### 2.6. Statistical Analysis 

Numerical variables, which were normally distributed, were described with means and standard deviations and with medians and interquartile ranges (IQR) in the case of non-normality. Categorical variables were described with frequencies and percentages. The association between gestation age and categorical outcome of BMD was examined by multinomial logistic regression. The association between gestation age and cut-off values of laboratory indicators of low BMD (pathological outcome of BMD examination) was examined by univariate logistic regression. The latter was also used to examine the association between certain risk factors and low BMD. The relationship between numerical variables was explored by Spearman correlation coefficient. The association between cut-off values of laboratory indicators and the categorical outcome of BMD was examined by the likelihood ratio test. Tests of relationships and associations, for which indicators of BMD were used, were based on values of the first measurement. The association between the categorical outcome of BMD at the first and the second measurement was examined by the marginal homogeneity test. All of the statistical tests were conducted at a significance level of α = 0.05. No correction for multiple testing was applied. Statistical analysis was performed using SPSS version 29.

## 3. Results

### 3.1. Maternal, Pregnancy-Related, and Children’s Characteristics 

Among infants born between 23 0/7 and 27 6/7, 42 subjects were eligible for this study.

This was the first delivery for more than half (23; 57.5%) of mothers included in the study. Two mothers (5%) had diabetes or primary hypertension, five (12.5%) had preeclampsia, and seven (17.5%) had chorioamnionitis. Prenatal steroids were given to 33 (82.5%) of the pregnant women. Thirteen women received vitamin D supplementation during pregnancy, while 16 did not. There was no data for 13 women out of 42.

The children’s characteristics and postnatal management are summarized in Table 1. 

Nutrition consisting of exclusively human milk with the addition of a human milk fortifier was given to 41 (97.6%) children, and fortification with one child was delayed due to enteral food intolerance (Table 1). Five (11.9%) children were parenterally nourished for more than 4 weeks. None of them were immobilized and the majority (34; 81%) had no infection. Thirty (71.4%) children developed bronchopulmonary dysplasia. The median length of hospitalization was 72.5 days (IQR: 61.8–86.5). None of the children died.

### 3.2. Laboratory Indicators of Metabolic Bone Disease

Laboratory indicators of MBD are summarized in Table 2. The absolute threshold value of total calcium was set at 2.04 mmol/L, ionized calcium at 1.2 mmol/L, and phosphate at 1.8 mmol/L. The values below that levels were considered low. The absolute threshold value of alkaline phosphatase was set at 10 µkat/L (600 IU/L). The values above that level were considered increased. No norms were set for bone-specific alkaline phosphatase and CTX-1.

### 3.3. Gestation Age and Bone Mineral Density 

There was a statistically significant association between gestation age and SOS value as the indicator of BMD. There was also a statistically significant correlation between CTX-1 and gestation age (r = 0.39; *p* = 0.012). However, no statistically significant association between gestation age and phosphate levels below 1.8 mmol/L, ALP above 600 IU/L (10 µkat/L), calcium below 2.04 mmol/L, and ionized calcium was found (Table 3).

### 3.4. Quantitative Ultrasound Measurement of the Tibial Bone Speed-of-Sound 

SOS values ranged from 2424 to 2949 m/s in the whole group of subjects at the 1st measurement. The mean for SOS at the 1st measurement was 2653.3 m/s and at the second 2735.5 m/s. The median was 2655 at the 1st and 2744.5 at the 2nd measurement. Supplementary Table lists the paired ultrasound measurements of BMD. 

There was no statistically significant correlation between quantitative laboratory indicators and SOS quantitative ultrasound measurements (Table 4).

We observed consistency between the two time-distant measurements in BMD categories in the same subject, although only the first was taken into account in the statistical calculation.

No statistically significant association between cut-off values of laboratory indicators of MBD and BMD measurements was found. CTX-1 was statistically significantly correlated with phosphate levels (r = 0.31; *p* = 0.045), but not with levels of alkaline phosphatase, calcium, or ionized calcium (Table 5). Bone-specific alkaline phosphatase was negatively and statistically significantly correlated with phosphate levels (r = −0.46; *p* = 0.002) and strongly and positively correlated with alkaline phosphatase (r = 0.93; *p* < 0.001).

### 3.5. Risk Factors and Bone Mineral Density 

According to manufacturer’s SOS value references and in terms of severity, BMD was categorized into two categories: the 1st with a Z-score of −1 to −2.5 and the 2nd with a Z-score < −2.5. None of the children within the 1st category received parenteral nutrition, and three (15%) children from the 2nd category received such nutrition (Table 6). The association was statistically significant (*p* = 0.048). One child (5.9%) categorized into the 1st group had late sepsis, while there were 11 (55%) children from the 2nd category having the same diagnosis. Children with late-onset sepsis had about 19 times higher odds (95% CI: 2.16–177.2) of having severely low BMD (Z-score < −2.5) than children who did not develop late-onset sepsis. No statistically significant association between other examined risk factors and low BMD was found. 

## 4. Discussion

Although this study confirmed an association between gestation age and quantitative ultrasound SOS value as an indicator of bone mineral density, the main finding was that bone turnover biochemical markers are less applicable as screening and diagnostic tools for MBD in preterm infants. To the best of our knowledge, this is one of the few studies to focus on the correlation between novel serum biochemical markers and quantitative ultrasonography for the identification of MBD in extremely premature newborns.

For adults, the World Health Organization defines osteopenia as a decrease in BMD by −1 to −2.5 Z score and osteoporosis as a decrease in BMD by more than 2.5 standard deviations below the average. The high burden of MBD among preterm infants was demonstrated by its presence in the majority (95%) of our study participants. The limit for abnormal BMD was set at—1 standard deviation, which is stricter than the definition for low BMD. Given the lack of consensus on its definition, the exact incidence of MBD is unknown, but it has been estimated to affect up to 60% of very low birth weight newborns [17]. The higher incidence can be explained by the fact that MBD was determined for all extremely premature infants, regardless of the presence of additional risk factors. Perhaps the incidence is underestimated and everyone should be screened.

MBD is mainly related to prematurity for the following reasons. The first is the lack of fetal mineral reserves due to premature birth. Since the majority (80%) of in utero calcium and phosphate transplacental accretion occurs during the third trimester, infants who are born preterm will be deprived of this mineral accumulation [17,18]. Notably, intrauterine growth retardation (IUGR) is associated with placental insufficiency, resulting in a higher reduction of active placental transport of minerals in utero. Although IUGR is an independent risk factor for low BMD [19], unlike the effect of low gestational age, it was not shown to have an important effect in our study group.

Due to incomplete data on vitamin D supplementation among the pregnant women in our study, there was no analysis of vitamin D in relation to bone mass in the offspring. Studies examining the aforementioned association remain inconclusive. Some research has shown no significant correlation between maternal vitamin D levels and bone parameters in newborns [20,21], while another [22] suggested a positive connection with offspring bone mass. For instance, vitamin D supplementation given to lactating mothers reduced vitamin D insufficiency, but there was insufficient evidence to evaluate its impact on vitamin D deficiency and bone health [22].

When enteral feedings were established, our subjects were fed exclusively with human milk, and most also with the addition of a human milk fortifier formula. While fortification of formula allows increased postnatal mineral delivery to premature infants, intestinal immaturity prevents optimal absorption [17,23]. Since the establishment of enteral feeding is often delayed or insufficient due to food intolerance and/or severe clinical course, parenteral supplementation is required. The findings of our study are consistent with a systematic review in which it was demonstrated that long-term parenteral nutrition (longer than 4 weeks) reduces bone mineral content in preterm individuals [24]. 

During the hospitalization of premature infants, due to their lung immaturity and the need for invasive mechanical ventilation, they can be more inactive due to immobilization and may lack motor stimulation, so there may be a risk of bone mineralization defects [25]. 

This is compounded by methylxantines, which may alter mineral levels [17], but this was not confirmed in our study participants, although methylxantines were used in 97.6% and diuretics in 11.9% of subjects.

Infection, particularly late-onset sepsis, is a common neonatal morbidity and can impair bone remodeling by reducing osteoblast proliferation, stimulating osteoclast activity, decreasing calcium absorption, and increasing calcium renal excretion [3,26]. In our study, children with late sepsis had about 19 times higher odds of MBD than children who did not develop late sepsis. Similar conclusions were reached in a study by Jensen [24], in which infants with sepsis and bronchopulmonary dysplasia were associated with an increased probability of MBD. In addition, the treatment of sepsis also prolongs the use of parenteral nutrition and increases the risk of MBD [19]. Hence, the reverse causality could also be attributed: newborns with low BMD were at higher risk for late-onset sepsis due to factors like nutritional deficiencies, lower gestational age, and prematurity-related illnesses, which can compromise their immune system and increase susceptibility to infections.

Quantitative ultrasonography (QUS) for identification of MBD

Neonatal bone quality, measured as density, is evaluated by various imaging tests. A dual-energy X-ray absorptiometry (DXA) scan is acknowledged to be the primary method for assessing whole BMD. However, its usefulness is limited for preterm infants because of the absence of standardized normative data for BMD. Additionally, its availability at the bedside is limited, and its usage involves exposure to ionizing radiation. The equipment itself tends to be large in comparison to the small bodies of ill preterms. Lastly, movement artifacts during scans is a frequent issue [17]. The other non-invasive method, quantitative ultrasound, offers distinct advantages over DXA scans. It is inexpensive, portable, and free of ionizing radiation, and it can provide information on BMD and the structure of the bone by measuring the speed of sound (SOS) [12,17]. Premature newborns with low BMD were identified by QUS and a significant association between gestation age and bone density was proven in our study. In a systematic review [11] of 29 studies assessing the use of QUS in preterm infants, the authors found a positive correlation between SOS values and gestational age and a postnatal decline in SOS values (more prominent fall noted in the more severe preterm infants). The consistency between two time-distant measurements in the same subject, although only the first was taken into account in the statistical calculation, can be stated as confirmation of the QUS method.

To assess overall mineral status, bone imaging is preferable to serum markers alone [27]. 

At 30–32 weeks of postmenstrual age, the BMD measurement is expected to be at its lowest. At that time, the physiological phenomenon of a relative decrease in BMD occurs due to a change in the proportions in newly formed and not yet mineralized bone [28,29]. Additionally, we expected MBD to be fully expressed at this age due to risk factors for extremely low birth weight. At term age, a BMD improvement was expected, but also bone formation and mineralization delays in comparison to term newborns.

Laboratory parameters for assessing MBD

There is no single precise biochemical marker that can be used for the diagnosis of MBD [27,30]. The most common biochemical screening tools for MBD include serum calcium, serum phosphate, alkaline phosphatase, parathormone, and 25-hydroxyvitamin D, but each test has limitations [17]. As there are a lack of reliable laboratory value references, especially in preterms [17], the results were given as the number (%) of subjects having laboratory values outside of the set absolute threshold values.

Blood calcium levels are regulated by calcitonin and parathormone. When the level of calcium in the blood decreases, it is mobilized from bone stores under the regulation of parathormone. Normal or high blood calcium is maintained in a calcium-deficient organism unless bone reserves are depleted in the late stage of MBD. Early MBD diagnosis via blood calcium is thus meaningless [19]. 

Hypophosphatemia signifies the earliest biochemical changes in MBD infants. Blood phosphorus levels serve as indicators of bone phosphate reserves, and a continual decrease suggests inadequate intake and heightened MBD risk. Persistent hypophosphatemia triggers increased bone resorption and escalating calcium excretion via the kidneys, leading to calcium depletion [19,31]. 

Variations in how the kidneys handle calcium and phosphorus can diminish the reliability of serum calcium and phosphorus levels as indicators of current mineral status, making them less reflective of underlying bone stores [27].

Furthermore, in the postpartum period after the detachment from the placenta and interruption of calcium flow, serum total and ionized calcium levels decrease, reaching a physiological nadir. The rate and extent of such a decline in calcium levels are inversely related to gestational age [32]. However, due to effective regulation of calcium homeostasis, levels soon do not differ from those of full-term infants. This fact was also confirmed among our subjects, in whom the levels of total or ionized calcium or phosphate were not associated with gestational age, nor was the level of calcium with MBD. 

Although the phosphate level itself was not associated with BMD in our study, there was a significant association between low phosphate (a “bone-mineralization ingredient”) and elevated levels of osteogenesis enzymes, such as bone-specific alkaline phosphatase and ALP. A combination of the criteria for “hungry bones syndrome (“serum total alkaline phosphatase activity above 900 IU/l” and “serum inorganic phosphate concentrations below 1.8 mmol/l”) has been suggested as a screening method for low BMD in preterms [33]. This was not confirmed in our study, nor in a study by Faerk [34], since BMD was not associated with the laboratory markers pair.

High metabolic demands for calcium and phosphate over the first few postnatal days lead to obligatory bone reabsorption. Alongside this process, there is also a rise in ALP activity aimed at enhancing bone mineralization in premature infants’ skeletons. When there is sufficient mineral substrate for mineralization, ALP levels tend to normalize. The bone may remain osteopenic for a longer period of time while it re-mineralizes, despite the normalization of ALP. Consequently, even if the overall mineral status remains inadequate, serum levels of phosphorus, calcium, and ALP may appear normal. 

The ALP range varies depending on age, gestational age [35], and sex; it is generally about 150–300 U/l in newborns [27], and it has been widely used as a marker for MBD, although there are no agreed cut-off values [27] and it is not a useful indicator of disease in isolation [15].

As with other authors, we found that ALP only achieves usefulness in a diagnostic and monitoring capacity when combined with other serum biomarkers and imaging [27].

Caution should be exercised in the interpretation of elevated ALP levels since these may be a symptom of hepatic and/or gastrointestinal diseases because this enzyme is also produced by the liver and gastrointestinal tract. An estimation of the bone isoenzyme of ALP is more specific for a skeletal cause and is suggested for diagnosing MBD [36]. However, reference ranges for bone ALP in a neonatal population are lacking. Furthermore, no association between either serum ALP or bone-specific alkaline phosphatase and gestational age and MBD was proven in our study.

The novel biomarkers of bone metabolism (PINP, bone formation marker) and CTX-I bone resorption marker are usually elevated in concert since resorption and formation are coupled processes. Due to their poor within-subject and between-lab reproducibility [37] and diurnal variation, none of them are routinely used as screening tools for MBD of prematurity. In our study, a significant correlation between CTX-I and gestation age was found, but no association with BMD or other biochemical markers. Due to technical problems with an inappropriate diluting reagent, PINP was not determined in the laboratory analysis, as initially intended.

Within the scope of our research, there was no correlation between serum biochemical markers and QUS for the identification of MBD in extremely premature newborns. The conclusion of a systematic review [30] was that it is difficult to establish the values of biochemical markers as early indicators of MBD.

Based on our study, no concordance with the applicability of QUS and laboratory parameters for assessing MBD was revealed.

An alternative for screening MBD in preterm infants includes tubular reabsorption of phosphate (TRP) and calcium (calcium/creatinine ratio). TRP measures the fraction of filtered phosphate reabsorbed by the kidney. It must be interpreted with serum phosphate levels at the same time. Low serum phosphate with high TRP suggests nutritional phosphate deficiency. Conversely, low TRP can indicate primary renal tubular damage or hyperparathyroidism, distinguishable by PTH levels. High PTH with low TRP suggests calcium deficiency, necessitating calcium supplementation to suppress elevated PTH levels. The urine calcium/creatinine ratio screens for hypercalciuria, often due to excess calcium intake or medications like loop diuretics or methylxanthines.

Decreased phosphate directly stimulates renal tubular synthesis of vitamin D, which increases intestinal calcium absorption. Thus, phosphate deficiency interferes with calcium balance, leading to hypercalcemia, hypercalciuria, and nephrocalcinosis [3]. Furthermore, in preterm infants, the low renal phosphate threshold, manifested by increased secretion of phosphorus in urine even when the value of serum phosphate is reduced, complicates the interpretation of urinary phosphate excretion [33,38].

The sensitivity and specificity of urinary calcium or urinary phosphate excretion tests are questionable [30,39], as urinary calcium and phosphate excretion vary due to influences like feeding type, immaturity, growth rate, and medications, making them unreliable for diagnosing MBD or determining supplementation needs [38].

However, the limitations of the study should also be considered, including the small sample size, undetermined ALP level defining the MBD of preterm infants at different gestational and neonatal ages, and unanalyzed PINP level due to technical problems with an inappropriate diluting reagent.

The utilization of the QUS method has some controversies. Factors like bone size and the specific site of measurement could significantly influence the accuracy of BMD measurements, particularly in growing children. Additionally, there is concern about the reliability of results obtained from a single BMD measuring site, as it may not provide a comprehensive reflection of the entire skeletal structure.

Studies [40] have shown that QUS measurements correlate moderately with DXA measurements of BMD in premature newborns. However, although DXA is less frequently used in this population due to concerns about radiation exposure, it represents the gold standard for BMD assessment.

The sensitivity and specificity of QUS in premature newborns are still under investigation, but initial studies suggest that it can effectively monitor bone development and detect early signs of MBD. However, due to the limited data, precise sensitivity and specificity values are not well established. Studies have shown that it can effectively identify individuals at higher risk of fractures, but it is not as precise as DXA in determining BMD [41,42].

QUS measures both bone mineral content and organic matrix [43]. The QUS parameters reflect bone density, microarchitecture, and elasticity, including bone mineralization and cortical thickness [41], providing a more complete picture of bone health as compared to current assessment techniques [11,42].

As QUS and DXA assess different bone tissue properties, occasional divergent results between them do not necessarily indicate methodological error [44] but emphasize the complementarity of the two methods [45].

Studies have shown that QUS can effectively identify individuals at higher risk for severe MBD, but it is not as precise as DXA in determining BMD. For a more accurate assessment of the importance of laboratory biomarkers, it would be reasonable to assess BMD with DXA, which we did not do due to all of the aforementioned limitations. Additionally, the impact of MBD severity on laboratory biomarkers could be inferred by comparison of the clinical characteristics of patients to laboratory biomarkers in our study.

## 5. Conclusions

While laboratory biomarkers of bone metabolism have proven to be less reliable in defining MBD, the complex interplay between them in premature infants should not be underestimated. Future research could focus on refining QUS techniques to improve accuracy and reliability, as well as exploring potential modifiers that may impact the relationship revealed between QUS and laboratory parameters. Despite the methodological advantages, risk factors should guide us in choosing who to screen.

## Figures and Tables

**Table 1:** Neonatal characteristics and postnatal management.

	n = 42
Gestation age; Median (IQR) (weeks)	26 (25–27)
Female sex (%)	17 (40.5)
Body mass (g); Mean (SD)	788 (206.7)
Intrauterine growth restriction (%)	13 (31)
Human milk + fortifier (%)	41 (97.6)
Human milk (unfortified) (%)	1 (2.4)
Parenteral nutrition (%)	5 (11.9)
Postnatal vitamin D (%)	42 (100)
Postnatal diuretics (%)	5 (11.9)
Postnatal methylxanthine (%)	41 (97.6)
Postnatal steroids (%)	19 (45.2)
Me (IQR) length of non-invasive ventilation (days)	22 (4.5–42.8)
Me (IQR) length of invasive ventilation (days)	8 (0–32.3)
Vasopressors (%)	1 (2.4)
Necrotizing enterocolitis (%)	4 (9.5%)
Early-onset sepsis (%)	12 (28.6%)
Late-onset sepsis (%)	15 (35.7%)
Systemic inflammatory response syndrome (SIRS) (%)	5 (11.9%)

**Table 2:** Laboratory indicators of metabolic bone disease.

Laboratory Indicator	1st Measurement Median (IQR)	1st Measurement Outside of the Absolute Threshold Values; n (%)	2nd Measurement Median (IQR)	2nd Measurement Outside of the Absolute Threshold Values; n (%)
Phosphate (mmol/L)	1.86 (1.56–1.99)	17 (40.5)	1.79 (1.54–1.94)	20 (52.6)
Alkaline phosphatase (IU/L)	9.91 (8.27–12.35)	19 (46.3)	10.44 (7.2–13.01)	6 (16.7)
Total calcium (mmol/L)	2.4 (2.3–2.47)	1 (2.4)	2.36 (2.31–2.42)	1 (2.6)
Ionized calcium (mmol/L)	1.37 (1.3–1.4)	0 (0)	1.36 (1.3–1.4)	0 (0)
Bone alkaline phosphatase (µg/L)	226.35 (176.7–288.6)		272 (178.9–338)	
CTX-1 (µg/L)	0.79 µg/L		0.66 (0.6–0.84)	

Abbreviation: CTX—C-terminal telopeptide type I collagen.

**Table 3:** Relationship between indicators of metabolic bone disease and gestation age (Spearman correlation coefficient).

Metabolic Bone Disease Indicators	Gestation Age
BMD (SOS value) (n = 37)	0.44 (*p* = 0.006)
Phosphate (n = 42)	0.28 (*p* = 0.075)
Alkaline phosphatase (n = 41)	−0.13 (*p* = 0.402)
Total calcium (n = 42)	0.19 (*p* = 0.236)
Ionized calcium (n = 39)	0.07 (*p* = 0.674)
CTX-1 (n = 42)	0.39 (*p* = 0.012)

Abbreviations: BMD—bone mineral density, SOS—speed-of-sound, CTX-1—C-terminal telopeptide type I collagen.

**Table 4:** Relationship between laboratory indicators of metabolic bone disease and quantitative ultrasound measurements of bone mineral density (Spearman correlation coefficient).

Laboratory Indicators	Bone Mineral Density (SOS-Values)
Phosphate (n = 37)	−0.02 (*p* = 0.886)
Alkaline phosphatase (n = 37)	0.04 (*p* = 0.816)
Total calcium (n = 37)	−0.08 (*p* = 0.650)
Ionized calcium (n = 36)	−0.15 (*p* = 0.387)
CTX-1 (n = 37)	0.07 (*p* = 0.670)

Abbreviations: SOS—speed-of-sound, CTX-1—C-terminal telopeptide type I collagen.

**Table 5:** Correlation between laboratory standard and novel indicators of metabolic bone disease (Spearman correlation coefficient).

Laboratory Indicators	CTX-1	Bone-Specific Alkaline Phosphatase
Phosphate (n = 42)	0.31 (*p* = 0.045)	−0.46 (*p* = 0.002)
Alkaline phosphatase (n = 41)	−0.21 (*p* = 0.187)	0.93 (*p* < 0.001)
Total calcium (n = 42)	0.01 (*p* = 0.935)	0.12 (*p* = 0.46)
Ionized calcium (n = 39)	−0.01 (*p* = 0.964)	0.01 (*p* = 0.956)

Abbreviations: CTX-1—C-terminal telopeptide type I collagen.

**Table 6:** Association between risk factors and bone mineral density (results of univariate logistic regression or the likelihood ratio test).

Risk Factors	Z-Score –1 to −2.5 (n = 17)	Z-Score < −2.5 (n = 17)	OR (95% CI)	*p*
Parenteral nutrition	0 (0)	3 (15)	-	0.048
Postnatal diuretics	2 (11.8)	2 (10)	0.83 (0.1–6.65)	0.863
Postnatal steroids	6 (35.3)	10 (50)	1.83 (0.49–6.9)	0.37
Necrotizing enterocolitis	0 (0)	2 (10)	-	0.11
Early sepsis	4 (23.5)	7 (35)	1.75 (0.41–7.45)	0.449
Late sepsis	1 (5.9)	11 (55)	19.56 (2.16–177.2)	0.008

## Data Availability

Data for this study are available upon reasonable request.

## Supplementary Materials

The following supporting information can be downloaded at: https://www.mdpi.com/article/10.3390/children11070784/s1, Figure S1: Paired quantitative ultrasound measurements of BMD-SOS values—at the post-menstrual age 30–32 weeks and 36–40 weeks; Table S1: Quantitative ultrasound measurements—speed-of-sound values (SOS values) at the post-menstrual age 30–32 weeks and 36–40 weeks.
