# Supplementary material for: Diagnostics of Metabolic Bone Disease in Extremely Preterm Infants—Clinical Applicability of Bone Turnover Biochemical Markers and Quantitative Ultrasound
Source: Children (Basel). 2024 Jun 27;11(7):784. doi: 10.3390/children11070784 (PMC11275004; doi:10.3390/children11070784)

Supplement

Table S1: Quantitative ultrasound measurements – speed-of-sound values (SOS values) at the post-menstrual age 30-32 weeks and 36-40 weeks.

Participant's number	1st measurement<br>Post-menstrual age 30-32 weeks	2nd measurement<br>Post-menstrual age 36-40 weeks
1	2611	NA
2	2531	2717
3	NA	2905
4	2652	2912
5	2757	2807
6	2775	2765
7	NA	2886
8	2776	2860
9	NA	3085
10	2763	2744
11	2688	2731
12	2739	3060
13	2627	2785
14	2551	2991
15	2633	2745
16	2655	2665
17	2645	2664
18	2620	2670
19	2443	2929
20	2424	2295
21	2718	2732
22	2870	2784
23	2780	2722
24	2760	2509
25	2910	2910
26	2667	2667
27	2502	2502
28	2472	2654
29	2707	2862
30	2930	2930
31	2745	2855
32	2947	2947
33	2768	2826
34	2717	2536
35	2946	2946

36	2458	2458
37	2350	2351
38	2324	2442
39	2578	2540
40	2453	2453
41	2531	2577
42	2661	NA
Mean	2653.3	2735.5
Median	2655	2744.5

Figure S1: Paired quantitative ultrasound measurements of BMD- SOS values- at the post-menstrual age 30-32 weeks and 36-40 weeks.

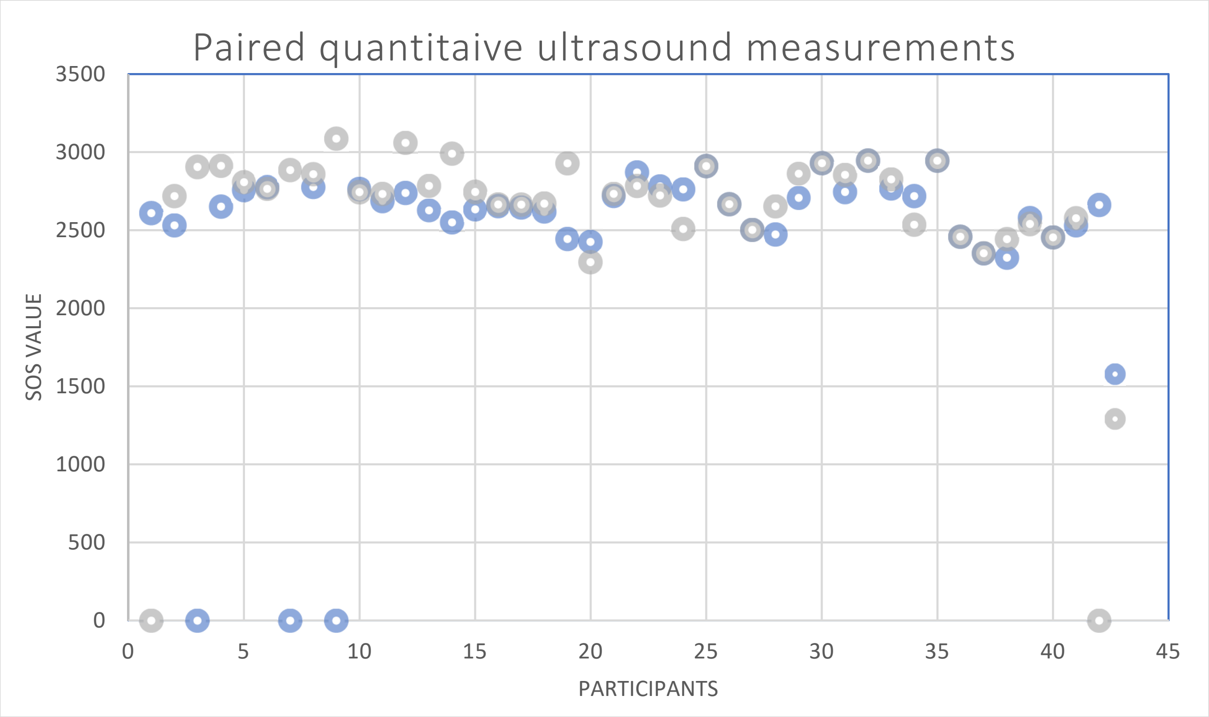

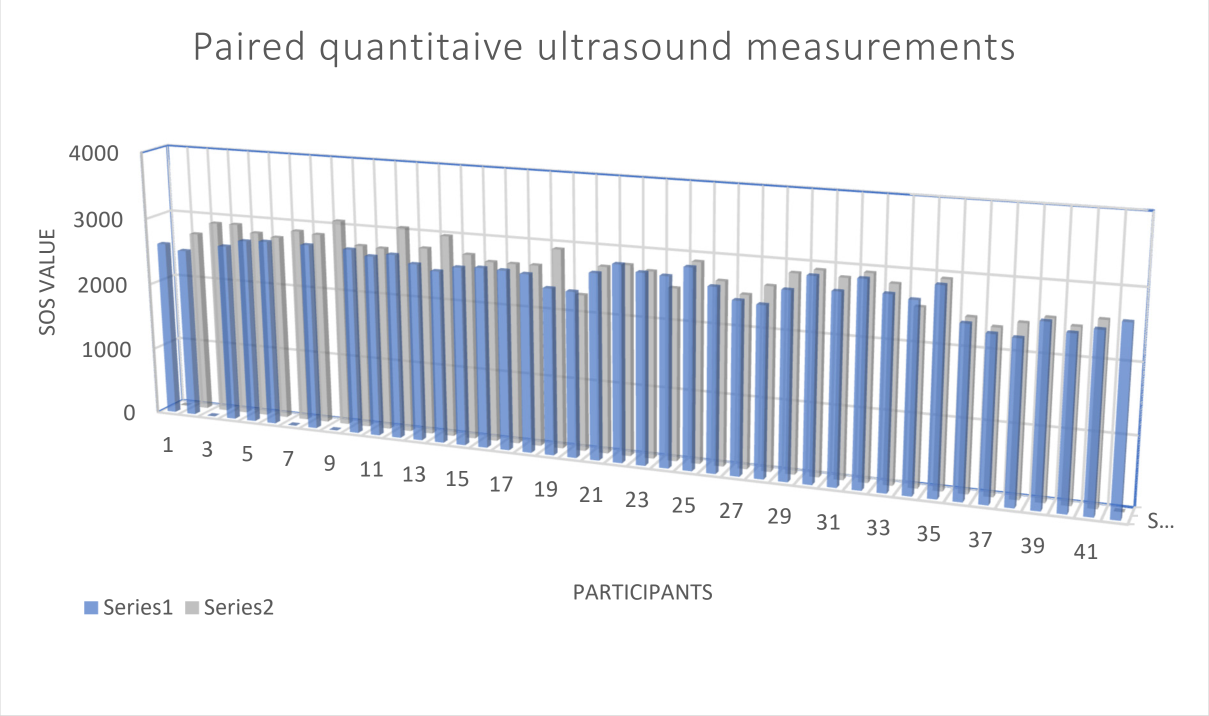

Supplement: Supplementary file 1 [file children-11-00784-s001.zip › children-3024209-supplementary.pdf]
